# Use of capture-based next-generation sequencing to detect ALK fusion in plasma cell-free DNA of patients with non-small-cell lung cancer

**DOI:** 10.18632/oncotarget.13741

**Published:** 2016-12-01

**Authors:** Shaohua Cui, Wei Zhang, Liwen Xiong, Feng Pan, Yanjie Niu, Tianqing Chu, Huimin Wang, Yizhuo Zhao, Liyan Jiang

**Affiliations:** ^1^ Department of Respiratory Medicine, Shanghai Chest Hospital, Shanghai Jiao Tong University, Shanghai, China

**Keywords:** liquid biopsy, anaplastic lymphoma kinase (ALK), capture-based next-generation sequencing, cell-free DNA (cfDNA), non-small-cell lung cancer (NSCLC)

## Abstract

Capture-based next-generation sequencing (NGS) is a potentially useful diagnostic method to measure tumor tissue DNA in blood as it can identify concordant mutations between cell-free DNA (cfDNA) and primary tumor DNA in lung cancer patients. In this study, the sensitivity, specificity and accuracy of capture-based NGS for detecting *ALK* fusion in plasma cfDNA was assessed. 24 patients with tissue *ALK*-positivity and 15 who did not harbor *ALK* fusion were enrolled. 13 *ALK*-positive samples were identified by capture-based NGS among the 24 samples with tissue *ALK*-positivity. In addition to *EML4-ALK*, 2 rare fusion types (*FAM179A-ALK* and *COL25A1-ALK*) were also identified. The overall sensitivity, specificity and accuracy for all cases were 54.2%, 100% and 71.8%, respectively. For patients without distant metastasis (M0-M1a) and patients with distant metastasis (M1b), the sensitivities were 28.6% and 64.7%, respectively. In the 15 patients who received crizotinib, the estimated median PFS was 9.93 months. Thus, captured-based NGS has acceptable sensitivity and excellent specificity for the detection of *ALK* fusion in plasma cfDNA, especially for patients with distant metastasis. This non-invasive method is clinically feasible for detecting *ALK* fusion in patients with advanced-stage NSCLC who cannot undergo traumatic examinations or have insufficient tissue samples for molecular tests.

## INTRODUCTION

Targeted molecular therapies are currently recommended as standard first-line treatments for patients with non-small-cell lung cancer (NSCLC) who harbor driver gene alterations. A driver gene recently validated in a subset of patients with NSCLC is the anaplastic lymphoma kinase (*ALK*) gene [1]. *ALK* gene rearrangements, which most often consist of a chromosome 2 inversion leading to fusion with the echinoderm microtubule-associated protein-like 4 (*EML4*) gene, are found in about 5% of NSCLC patients [2, 3]. In patients with *ALK*-positive NSCLC, crizotinib, an adenosine triphosphate (ATP)-competitive, small-molecule, multi-targeted tyrosine kinase inhibitor (TKI), has demonstrated promising efficacy in many clinical studies, both in first-line and non–first-line settings [3–5].

Molecular pathological examinations are necessary before administering targeted therapies as first-line therapies in unselected patients with NSCLC [6]. For *ALK* detection, the Ventana ALK(D5F3) immunohistochemistry (IHC) test and fluorescence *in situ* hybridization (FISH) are commonly used in current clinical practice. These two methods have been shown to provide concordant results for *ALK* detection [7, 8]. Although genotyping tumor biopsies for targetable somatic alterations has become routine clinical practice for NSCLC, many concerns remain about the results of tumor tissue samples being considered the ‘gold standard’. Firstly, the quality and quantity of the available tumor biopsy or cytology material is not always adequate to perform these essential molecular tests since most patients with NSCLC are initially diagnosed with advanced disease [9]. Secondly, dynamic detection, which often requires repeated tumor biopsies, is needed to monitor genetic evolutions and discover possible acquired molecular mechanisms, and this is mostly impossible clinically [10]. Moreover, serial biopsies are invasive examinations and may be technically difficult (eg, for tumor-containing blood vessels or where necrosis exists) and could involve serious risks for patients, especially those with a poor performance status [11].

The non-invasive ‘liquid biopsy’ method is an emerging technology that has the potential to make up for the above limitations [12]. Recently, circulating cell-free DNA (cfDNA) isolated from blood samples has been shown to contain genetic alterations representative of those detected in the primary tumor tissue DNA [10]. cfDNA consists predominantly of fragmented DNA released into the circulation by tumor cells undergoing apoptosis. Next-generation sequencing (NGS), also known as massively parallel sequencing, is becoming a potentially useful diagnostic method for future clinical practice application as it is able to accurately detect most genomic alterations in a single assay [13–15]. Studies using capture-based NGS to detect cfDNA in patients with lung cancer have identified concordant mutations between cfDNA and primary tumor DNA, suggesting that this technique may be a practical diagnostic method for measuring tumor tissue DNA in blood. However, the feasibility and performance of capture-based NGS for detecting the *ALK* gene has not been extensively studied to date. Therefore, in the present study, we collected blood samples from patients with NSCLC treated at our institute, and analyzed the *ALK*-fusion status in plasma cfDNA by using capture-based NGS.

## RESULTS

### Patient characteristics

The demographic and clinicopathologic characteristics of the 39 patients enrolled in the study are shown in Table 1. Patients with tissue *ALK*-positivity (*n* = 24) tended to be young (mean age 51 years; range 31 to 68 years), male, never smokers, and have advanced clinical disease (stages IIIB/IV). All *ALK*-positive patients had a histological type of adenocarcinoma. *ALK*-negative patients had a mean age of 60 years (range 47 to 73 years), and were more likely to be male, never smokers, and have an adenocarcinoma histological type.

**Table 1:** Demographic and clinicopathologic characteristics of the 39 patients enrolled in the study

Characteristics	Total [*N* = 39] *n* (%)	*ALK*-positive (tissue) [*N* = 24] *n* (%)	*ALK*-negative (tissue) [*N* = 15] *n* (%)
Age, years:			
Mean	55	51	60
Range	31–73	31–68	47–73
Sex:			
Male	27 (69)	18 (75)	9 (60)
Female	12 (31)	6 (25)	6 (40)
Histological type:			
Adenocarcinoma	37 (95)	24 (100)	13 (87)
Squamous cell	2 (5)	0	2 (13)
Smoking history:			
Never smokers	25 (64)	17 (71)	8 (53)
Ever Smokers	14 (36)	7 (29)	7 (47)
Clinical stage:			
IB-IIIA	7 (18)	2 (8)	5 (33)
IIIB-IV	32 (82)	22 (92)	10 (67)
Family cancer history:			
Yes	8 (21)	4 (17)	4 (27)
No	31 (79)	20 (83)	11 (73)

### Performance of the NGS method for *ALK* detection in plasma samples

**Figure 1:**
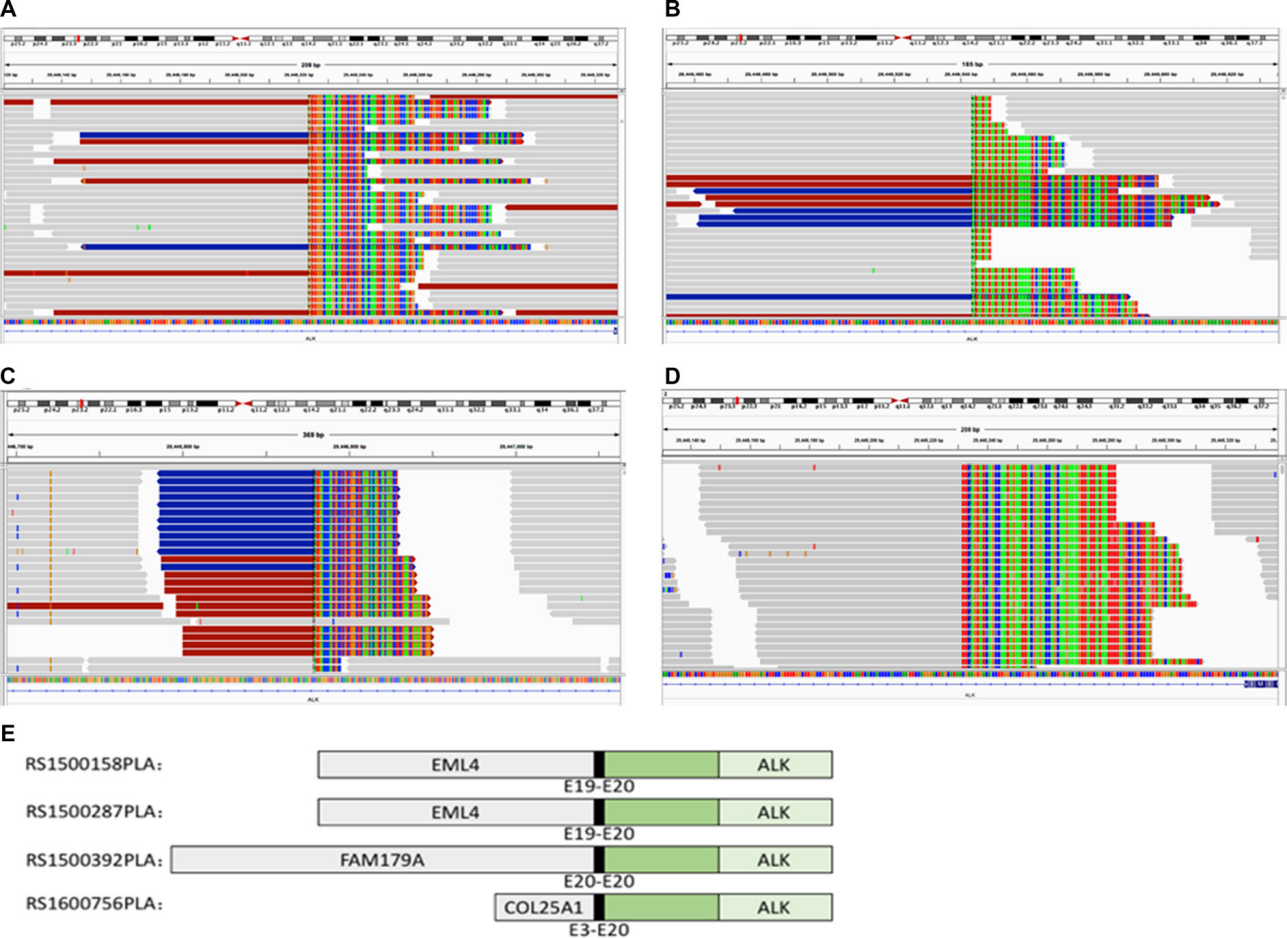
Integrative Genomics Viewer (IGV) screenshot showing that the breakpoints on the ALK gene detected by capture-based next-generation sequencing were identical among different blood samples (**A** and **B**) The IGV screenshot of *EML4-ALK* rearrangement. (**C**) The IGV screenshot of *FAM179A-ALK* rearrangement. (**D**) The IGV screenshot of *COL25A1-ALK* rearrangement. (**E**) The schematic diagram of ALK fusion proteins. The green region signifies the tyrosine kinase domain.

**Figure 2:**
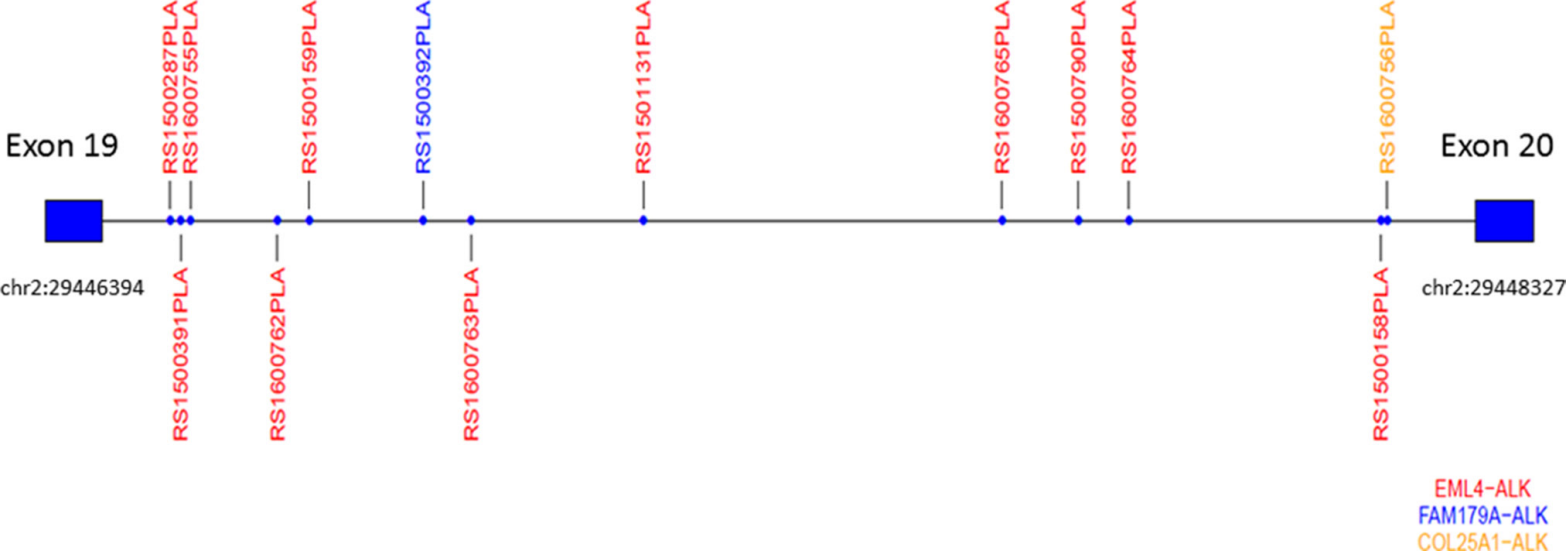
The distribution of the breakpoints on ALK intron 19 identified by capture-based next-generation sequencing

With the use of capture-based NGS, we identified 13 *ALK*-positive samples among the 24 tissue samples with *ALK*-positivity. In addition to the common fusion type *EML4-ALK* (Figure 1A and 1B), capture-based NGS also identified 2 rare fusion types: *FAM179A-ALK* (Figure 1C) and *COL25A1-ALK* (Figure 1D). A schematic diagram of the ALK fusion proteins is shown in Figure 1E. The breakpoints of the *ALK* gene were all located on *ALK* intron 19. The distribution of the breakpoints on *ALK* intron 19 detected by capture-based NGS is shown in Figure 2.

Sensitivity, specificity and accuracy data for the capture-based NGS method are shown in Table 2. The overall sensitivity for all cases was 54.2%. For patients with earlier clinical stages (stages IB to IIIA) and those with an advanced stage (stages IIIB/IV), the sensitivities were 50% (1/2) and 54.5% (12/22), respectively (*P* = 1.000; data not given in Table 2). For patients without distant metastasis (M0-M1a) and those with distant metastasis (M1b), the sensitivities were 28.6% (2/7) and 64.7% (11/17), respectively (*P* = 0.182; data not given in Table 2).

**Table 2:** Performance assessment of next-generation sequencing methods for *ALK* detection using plasma samples

Tissue (‘gold standard’)		*ALK+*	*ALK+*	*ALK–*	*ALK–*
Plasma cfDNA (NGS)		*ALK+*	*ALK–*	*ALK+*	*ALK–*
Total (*N* = 39)	n	13	11	0	15
	Sensitivity	54.2%			
	Specificity	100%			
	Accuracy	71.8%			
Stage IB to IIIA (*N* = 7)	n	1	1	0	5
	Sensitivity	50%			
	Specificity	100%			
	Accuracy	85.7%			
Stage IIIB/IV (*N* = 32)	n	12	10	0	10
	Sensitivity	54.5%			
	Specificity	100%			
	Accuracy	68.8%			
M0+M1a (*N* = 16)	n	2	5	0	9
	Sensitivity	28.6%			
	Specificity	100%			
	Accuracy	68.8%			
M1b (*N* = 23)	n	11	6	0	6
	Sensitivity	64.7%			
	Specificity	100%			
	Accuracy	73.9%			

cfDNA, cell-free DNA; NGS, next-generation sequencing.

All *ALK*-negative tissue samples were detected as negative results using the capture-based NGS method. The specificity of capture-based NGS was 100%. For all cases, the accuracy was 71.8%, and for patients with early and advanced clinical stages the accuracy was 85.7% and 68.8%, respectively (*P* = 0.649; data not given in Table 2). The accuracy for M0-M1a and M1b patients was 68.8% and 73.9%, respectively (*P* = 0.734; data not given in Table 2).

### Progression-free survival (PFS) data in patients who received crizotinib

**Figure 3:**
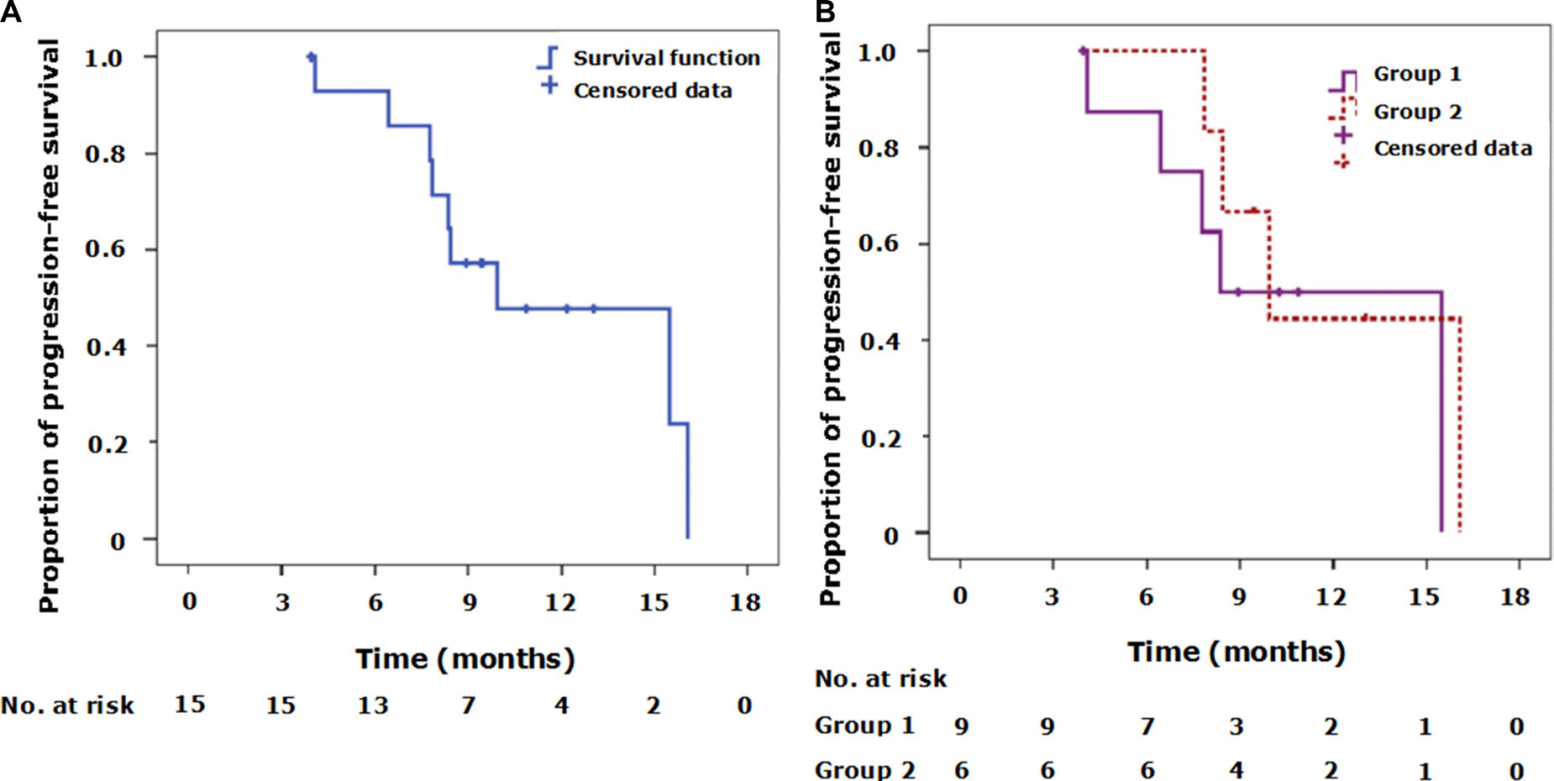
Kaplan-Meier curves of progression-free survival (PFS) for the 15 patients who received crizotinib therapy (**A**) The Kaplan-Meier curve of progression-free survival for all patients. Tick marks represent censored observations. (**B**) Kaplan-Meier curves of progression-free survival for 2 groups of patients with different plasma *ALK* detection results. Group 1 includes patients with tissue *ALK*-positivity and plasma *ALK*-positivity. Group 2 includes patients with tissue *ALK*-positivity but plasma *ALK*-negativity. No statistically significant differences in PFS were observed between Group 1 and Group 2. Tick marks represent censored observations.

Of the 24 *ALK*-positive patients, 15 (62.5%) received crizotinib therapy. Nine of the 15 patients (60%) had occurred progressive disease (PD) at the study cutoff date. The estimated median PFS for all 15 patients was 9.93 months (95% CI 4.10–15.78 months; Figure 3A). The 2 patients harboring rare *ALK* fusion types both received first-line crizotinib therapy. The patient harboring *COL25A1-ALK* had PD after treatment with crizotinib for about 6 months, but the patient harboring *FAM179A-ALK* had not progressed at the study cutoff date. The PFS for the patients harboring *FAM179A-ALK* and *COL25A1-ALK* fusion types were 12.06 months and 6.28 months, respectively (Table 3).

**Table 3:** Clinicopathologic characteristics and outcomes of crizotinib therapy in 2 patients with rare *ALK* fusion types detected by capture-based NGS

Fusion type	Sex	Age (years)	Histology	Surgical history	Clinical stage before crizotinib therapy	ag	Crizotinib therapy			
							Timing	Overall response	Status	PFS (months)
*FAM179A-ALK*	Female	36	Adenocar- cinoma	None	T4N2M1b IV	None	First-line	PR	Not PD	12.06
*COL25A1-ALK*	Male	58	Adenocar-cinoma	Resected P-T2aN0M0 Poorly differ- entiated	r-T0N0M1b IV	None	First-line	PR	PD	6.28

NGS, next-generation sequencing; PD, progressive disease; PFS, progression-free survival; PR, partial response.

The 15 patients treated with crizotinib were divided into 2 groups according to their detection results. Group 1 included patients with tissue *ALK*-positivity and plasma *ALK*-positivity, while Group 2 included those with tissue *ALK*-positivity and plasma *ALK*-negativity. The demographic and clinicopathologic characteristics of these 2 groups of patients are summarized in Table 4. The estimated median PFS values were 8.35 months (95% CI 4.07–12.62 months) for Group 1, and 9.93 months (95% CI 6.97–12.90 months) for Group 2. However, no statistically significant differences between the groups were observed using a 2-sided log-rank test (*P* = 0.482; Figure 3B).

**Table 4:** Demographic and clinicopathologic characteristics of 2 groups of patients with differing plasma ALK detection results who were treated with crizotinib

Characteristics	Group 1 [*N* = 9] Tissue*ALK+*/Plasma*ALK+* *n* (%)	Group 2 [*N* = 6] Tissue*ALK+*/Plasma*ALK−* *n* (%)	*P*-value
Age:			
< 60 years	7 (78)	4 (67)	1.000
≥ 60 years	2 (22)	2 (33)	
Sex:			
Male	6 (67)	4 (67)	1.000
Female	3 (33)	2 (33)	
Histological type:			
Adenocarcinoma	9 (100)	6 (100)	1.000
Squamous cell	0	0	
Smoking history:			
Never smokers	9 (100)	4 (67)	0.143
Ever Smokers	0	2 (33)	
Clinical stage:			
IIIB	0	3 (50)	0.044*
IV	9 (100)	3 (50)	

* *P* < 0.05.

## DISCUSSION

This study analyzed the *ALK*-fusion status in plasma cfDNA with capture-based NGS. By using the results of tissue *ALK* detection as the ‘gold standard’, we found that the sensitivity of capture-based NGS for all 39 patients studied was more than 50%, but that sensitivity varied in patients with different clinical stages. The sensitivity of the method was higher in patients who had distant metastasis than in those who did not, although the difference was not statistically significant. Importantly, the specificity of capture-based NGS was 100% as all tissue samples with *ALK*-negativity were also detected as *ALK*-negative using the NGS method. When patients who received crizotinib were divided into 2 groups according to the NGS results, we did not observe any significant differences in PFS between NGS-positive and NGS-negative groups.

Consistent with previously reported results [16–18], our study indicated that patients who were *ALK*-positive were predominantly young, never smokers, and had an adenocarcinoma histology. The occurrence of *ALK* gene rearrangements, which are present in about 5% of patients with NSCLC, is relatively rare compared with epidermal growth factor receptor (EGFR) mutations [1, 19]. *EML4-ALK* is the most common *ALK* fusion types identified in NSCLC [2, 3]. Capture-based NGS is able to provide detailed information on the arrangements detected, including the fusion partner and the precise location of the breakpoints on both genes, which cannot be determined by traditional IHC or FISH methods. In addition to *EML4-ALK* rearrangement, capture-based NGS also identified 2 rare *ALK* rearrangement types in this study, *FAM179A-ALK* and *COL25A1-ALK*, and the breakpoints of the *ALK* gene were all located on *ALK* intron 19 with all 3 fusion types.

To assess the performance of capture-based NGS, we evaluated data in all patients enrolled in the study and in subgroups according to the patients’ clinical stages. We found that the consistency of capture-based NGS for *ALK* detection in plasma cfDNA was 71.8% in all patients regardless of clinical stages, with a sensitivity of 54.2% and a specificity of 100%. This sensitivity value differs from that reported in a recent study evaluating the performance of deep NGS for plasma *ALK* detection [20], which reported a sensitivity of 77.8%. However, the sample size of this study was only 18, and all of the patients were defined as having advanced clinical stages [20]. In contrast, 2 patients (8.3%, 2/24) with relatively early clinical stages were included in our sensitivity analysis.

As previous reported, the clinical stage can influence the detection sensitivity. In a study evaluating the diagnostic accuracy of detecting *EGFR* in lung cancer by deep sequencing of plasma cfDNA, the overall sensitivity was 54.4% for all cases [21]. However, sensitivities for patients with stages IA-IIIA and patients with stages IIIB-IV disease were 22.2% and 72.7%, respectively [21]. Another study also found that the clinical stage significantly affected detection sensitivities: the sensitivity was 10% in early-stage patients and 56% in advanced-stage patients (*P* = 0.0014) [22]. However, we found no obvious differences in the sensitivity of capture-based NGS between patients with early stage disease (stage IB-IIIA) and those with advanced stages (stages IIIB-IV). On the other hand, when the patients were divided into M0-M1a and M1b subgroups, a difference in the sensitivity was observed (28.6% for the M0-M1a subgroup versus 64.7% for the M1b subgroup), although this difference was not statistically significant. However, the limited sample size of the 2 subgroups may have biased this result. Specific reasons for different sensitivities for plasma *ALK* detection between patients with different clinical stages remain unknown. It is possible that patients with advanced clinical stages release more cfDNA into the blood so that it can be more easily detected compared with patients with earlier clinical stages. In support of this hypothesis, cfDNA concentrations have been found to be significantly associated with tumor stage [23].

The implications of the sensitivity assessment of capture-based NGS for clinical practice are worth considering. cfDNA in plasma has the potential to be used as a surrogate for tumor tissues for detecting genetic alterations [24]. As the sensitivity of capture-based NGS was low in patients with early-stage NSCLC (especially those with M0-M1a disease according to our results), capture-based NGS may be preferred for advanced NSCLC. However, this should not limit its use as patients with early stage NSCLC are not usually recipients of ALK-TKI therapy. In addition, only patients with metastatic NSCLC are recommended *ALK* gene testing by the National Comprehensive Cancer Network (NCCN) guidelines.

An important finding of our study was that the specificity of capture-based NGS for plasma *ALK* detection was 100%, which means that false-positive results are unlikely. Clinically, the high specificity of capture-based NGS may enable the recommendation of ALK-TKI therapy on the basis of positive results with plasma cfDNA testing.

Among the patients who received crizotinib in this study, we found that the median PFS of those with plasma *ALK*-negativity (Group 2) was longer than that of patients with plasma *ALK*-positivity (Group 1), although the difference was not statistically significant. While bias resulting from the small sample size needs to be taken into account when considering this finding, we consider that the clinical stage may play a role as the *ALK*-negative group (which comprised 6 patients) included 3 with stage IIIB disease, while the *ALK*-positive group (which comprised 9 patients) all had stage IV disease (*P* = 0.044; Table 4).

For the 2 patients in whom rare *ALK* rearrangement types (*FAM179A-ALK* and *COL25A1-ALK*) were detected by capture-based NGS, we observed that both had a partial response with crizotinib therapy. Although the patient with the *FAM179A-ALK* fusion type had not progressed at the study cutoff date, the patient with the *COL25A1-ALK* had PD after 6 months of crizotinib therapy. Further studies are needed to determine whether there are differences in the efficacy of ALK-TKI therapy between patients with common fusion types (*EML4-ALK*) and those with rare *ALK* fusion types (eg, *FAM179A-ALK* and *COL25A1-ALK* in this study).

This study has some limitations. Firstly, the sample size was relatively small due to the low incidence of *ALK* fusion among patients with NSCLC. Secondly, it was a single center study with insufficient representability. Therefore, its results should be interpreted with caution as they need to be confirmed by subsequent multicenter studies with larger sample sizes. Thirdly, dynamic detection, which is considered one of the most important advantages of plasma samples, was not reflected in the present study due to its design.

In conclusion, capture-based NGS has an acceptable sensitivity for detecting *ALK* fusion in plasma cfDNA, especially for patients with distant metastasis. Importantly, the specificity of capture-based NGS was 100%, which means that a true-positive *ALK* status can be diagnosed by *ALK*-positivity with this method. In clinical practice, the non-invasive method is clinically feasible for the detection of *ALK* fusion in patients with advanced-stage NSCLC who cannot undergo traumatic examinations or who have insufficient tissue samples for molecular tests.

## MATERIALS AND METHODS

### Patient selection criteria and sample preparation

Twenty-four patients with *ALK*-positivity (identified by the Ventana ALK(D5F3) IHC anaysis and FISH) and 15 who did not harbor *ALK* fusion were prospectively enrolled in the study at the Shanghai Chest Hospital, Shanghai Jiao Tong University between March 31, 2015 and February 18, 2016. All patients were histologically diagnosed as NSCLC. Patients with symptomatic brain metastases or an Eastern Cooperative Oncology Group performance status (ECOG PS) of more than 2 were excluded, but there was no limit to the clinical stage for selection. Prior to enrollment, tissue samples were detected for *ALK* mutations. For each patient who met the selection criteria, 10 mL samples of peripheral blood samples were collected.

The study was approved by Ethics Committee of Shanghai Chest Hospital, Shanghai Jiao Tong University, Shanghai. All patients provided written informed consent to participate in the study prior to enrollment.

### *ALK* detection in tissue samples

Tumor tissue samples obtained by either diagnostic or surgical procedures were used for *ALK* mutation detection. We used an IHC analysis which was conducted with the monoclonal antibody D5F3 (Ventana Medical Systems, Tucson, AZ, USA) to screen for *ALK*-positivity. FISH was used to confirm the outcome of the immunohistochemical analysis when it could not be defined. The positive cutoff value of FISH was defined as 15%.

### *ALK* detection in blood samples

### Preparation of plasma cell-free DNA

10 mL blood samples were collected in K3EDTA-containing tubes (Cell-Free DNA BCT) and centrifuged at 2000 g for 10 minutes at 4°C within 72 hours of their collection. The carefully aspirated plasma supernatant was transferred into fresh 15 mL centrifuge tubes without disturbing the buffy coat layer. The plasma samples were then centrifuged for 10 min at 16,000 g at 4°C and the supernatant was removed to a new tube with a pipette without disturbing the pellet. The plasma was stored at −80°C until further analysis. Circulating cfDNA was recovered from 4 to 5 mL of the plasma samples using the QIAamp Circulating Nucleic Acid kit (Qiagen).

### Quantification of plasma cell-free DNA

Quantification of cfDNA was performed using the Qubit 2.0 Fluorometer with dsDNA HS assay kits (Life Technologies, Carlsbad, CA, USA). The starting material for subsequent testing consisted of 50 ng cfDNA prepared by the Qiagen method.

### Capture-based targeted DNA sequencing

DNA was profiled using a commercially available capture-based sequencing panel - LungPlasma panel (Burning Rock Biotech Ltd, Guangzhou China) targeting 168 genes and spanning 160K of human genomic regions. DNA was hybridized with the capture probe baits, selected with magnetic beads, and polymerase chain reaction (PCR)-amplified. A bioanalyzer high sensitivity DNA assay was then used to assess the quality and size range, and 30 indexed samples were sequenced onto a NextSeq 500 (Illumina, Inc., USA) with pair-end reads. The details of the LungPlasma panel was shown in Supplementary Table S1.

### Sequence data analysis

Sequence data were mapped to the human genome (hg19) using a BWA aligner 0.7.10. Local alignment optimization was performed using GATK 3.2 with default parameters. Variant calling was performed using MuTect with default parameters, and VarScan with parameters (−min-coverage = 8, −min-reads = 2, −min-avg-qual = 15, −min-var-freq = 0.01 −strand-filter = 1, −*P*-value = 0.99). DNA translocation analysis was performed using both Tophat2 and Factera 1.4.3 with parameters (-C -F -r 5 -m 2 -k 8 -c 12).

### Crizotinib administration and response evaluation

Fifteen patients in the study cohort received crizotinib therapy. Before initiation of crizotinib, all patients were evaluated by computed tomography (CT) of the thorax, bone scanning, enhanced magnetic resonance imaging (MRI) of the brain, and abdominal ultrasound. In addition, routine hematology tests (eg, red blood cell, hemoglobin, white blood cell, and platelet counts), biochemistry analyses (including alanine aminotransferase, aspartate aminotransferase, alkaline phosphatase, glutamyl endopeptidase, and lactate dehydrogenase), coagulation tests, and urinalyses.

Crizotinib was administered orally in a dosage of 250 mg twice daily in 28-day cycles. The tumor response was assessed after the first cycle of crizotinib therapy and subsequently after every 2 cycles according to RECIST (Response Evaluation Criteria in Solid Tumors), version 1.1. CT scans were used to assess the response to crizotinib when clinically indicated or until discontinuation of crizotinib treatment. The overall response to crizotinib was documented as either a complete response (CR), partial response (PR), stable disease (SD), or PD. The cutoff date for the study was October 31, 2016.

### Statistical analysis

The performance of captured-based NGS for plasma *ALK* detection was assessed by determining its sensitivity, specificity and accuracy using the tissue detection results as the ‘gold standard’. Sensitivity is the probability that captured-based NGS will indicate *ALK*-positivity among patients with tissue *ALK*-positivity, while specificity is the fraction of those with tissue *ALK*-negativity who have a negative test result using NGS. Accuracy is defined as the probability that captured-based NGS will indicate the same situations as the tissue results in all samples.

Two-sided Fisher's exact tests were used for analyzing the results. PFS was calculated as the time from the date crizotinib was first administered until the date of objective PD, according to RECIST version 1.1. The Kaplan-Meier method was used to estimate PFS, and 2-sided log-rank tests were applied to compare differences between treatment groups. *P*-values less than 0.05 were considered statistically significant. The survival analyses were performed using SPSS^®^ software, version 13.0 (SPSS Inc., Chicago, IL, USA).

## SUPPLEMENTARY MATERIALS TABLE





## Abbreviations

ALK: anaplastic lymphoma kinase
ATP: adenosine triphosphate
cfDNA: cell-free DNA
CR: complete response
CT: computed tomography
ECOG PS: Eastern Cooperative Oncology Group performance status
EGFR: epidermal growth factor receptor
EML4: echinoderm microtubule-associated protein-like 4
IHC: immunohistochemistry
FISH: fluorescence *in situ* hybridization
IGV: Integrative Genomics Viewer
MRI: magnetic resonance imaging
NGS: next-generation sequencing
NSCLC: non–small-cell lung cancer
PCR: polymerase chain reaction
PD: progressive disease
PFS: progression-free survival
PR: partial response
RECIST: Response Evaluation Criteria in Solid Tumors
SD: stable disease
TKI: tyrosine kinase inhibitor.
