# Supplementary material for: Use of capture-based next-generation sequencing to detect ALK fusion in plasma cell-free DNA of patients with non-small-cell lung cancer
Source: Oncotarget. 2016 Dec 1;8(2):2771–80. doi: 10.18632/oncotarget.13741 (PMC5356840; doi:10.18632/oncotarget.13741)
Supplement: Supplementary file 1 [file oncotarget-08-2771-s001.pdf]

Use of capture-based next-generation sequencing to detect *ALK* fusion in plasma cell-free DNA of patients with non-small-cell lung cancer

Supplementary Materials

Supplementary Table S1: The hybrid capture panel (LungPlasma panel) used to detect ALK fusion. see Supplementary_Table_S1
